# Influence of information, education and communication on prenatal and skilled delivery in the Tano North District, Ghana: A cross-sectional study

**DOI:** 10.1016/j.heliyon.2021.e07245

**Published:** 2021-06-08

**Authors:** Evelyn Amponsah, Adam Fusheini, Awolu Adam

**Affiliations:** a Department of Epidemiology and Biostatistics, School of Public Health, University of Health and Allied Sciences, Ho, Ghana; b Ghana Health Service, Disease Control Unit, Tano North District, Ahafo Region, Ghana; c Department of Preventive and Social Medicine, Otago Medical School, University of Otago, Dunedin, New Zealand; d Department of Family and Community Health, School of Public Health, University of Health and Allied Sciences, Ho, Ghana; e Center for Health Literacy and Rural Health Promotion, Accra, Ghana

**Keywords:** Information, Education and communication, Prenatal and skilled delivery, Birth attendants, Ghana

## Abstract

Skilled birth attendance is considered an effective intervention to reduce maternal and early neonatal morbidity and mortality. Yet in Ghana, skilled birth attendance is said to be relatively low despite high antenatal attendance. In this study, we specifically assessed the influence of information, education and communication on prenatal and skilled delivery in the Tano North District of Ghana. A descriptive cross-sectional quantitative survey involving both closed and open-ended questionnaires were conducted among 393 women at three health facilities. The results showed skilled health personnel attended 94.1% of deliveries, which is higher than what has been reported in previous studies. Mothers with Senior High School Education were found to be 11.46 times more likely to be delivered by skilled birth attendant than those without formal education COR = 11.46, 95% (2.01–65.19) and this was statistically significant p = 0.006. There was also a significant association between information received by pregnant women and place of delivery (X^2^ = 20.85, P = 0.000 α = 0.05) in that the usefulness of information to mothers influenced their choice of health facility delivery. Marital status was also strongly correlated to being attended by skilled birth attendant (χ2 = 14.73, p = 0.005) as 0.4 times of mothers who were married were more likely to be attended by skilled birth attendants as compared to those that are single. COR = 0.005, 95% CI (0.00–0.36) and this was statistically significant p = 0.002. This study suggests the incorporation of IE&C into nursing training curriculum to orient students on the importance of IE&C so as to improve ANCs and skilled birth attendance further.

## 1 Introduction

The proportion of births attended by skilled health professionals presents opportunities for preventing some maternal and child deaths as it ensures clean and safe delivery practices (Amoah et al., 2016). Health facility-based delivery with skilled health professionals guarantees women's and the expected children's health, which constitutes a prime concern to public health discourse (Amoah et al., 2016). It is in the light of this that the World Health Organisation (WHO) identified Antenatal Care (ANC) as an indicator of maternal health with greater potential to reduce maternal mortality ratios (Dickson et al., 2018; WHO, 2006). Maternal mortality ratio (MMR) remains a worldwide public health concern as about 295,000 women died during and following pregnancy and childbirth in 2017; translating to approximately 810 women dying from preventable causes related to pregnancy and childbirth every day in 2017 (WHO, 2019a). The vast majority of these deaths (94%) occurred in low-resource settings of Sub-Saharan Africa and Southern Asia (WHO, 2019a). Approximately 86% (254,000) of the estimated global maternal deaths in 2017 occurred in the two regions with Sub-Saharan Africa accounting for roughly two-thirds (196,000) of the maternal deaths, while Southern Asia accounted for nearly one-fifth (58,000) (WHO, 2019a). In Ghana, the setting of this study, the maternal mortality ratio currently stands at 308 deaths per 100,000 live births [223-420-uncertainty interval] (Konlan et al., 2020; WHO, 2019b, 2019c) though this figure is relatively lower compared to the number of maternal deaths that occurred over a decade ago (Konlan et al., 2020; WHO, 2019c). Skilled care before, during and after childbirth could prevent these deaths thereby saving the lives of women and new-borns (WHO, 2019a). One key way is by antenatal care and skilled birth attendance. ANC is defined as “a public health service with the goal of preventing health risks, early detection of abnormalities, institution of corrective measures if possible and preparation of both the woman and foetus and to ensure good start of life for each newborn child” (Manyeh et al., 2020). Pregnant women and their families receive advice and information on health promotion, and preventive health services including the management of a healthy pregnancy, safe childbirth, nutritional support, early exclusive breast feeding and encouraging assisted delivery (Dickson et al., 2018) through ANC.

The 2030 Agenda for Sustainable Development highlights the importance of continued attention to maternal and newborn health (MNH) under Sustainable Development Goal 3 (WHO, 2018). This emphasises the significance of skilled delivery or skilled birth attendance defined as proportion of births attended by skilled health personnel (WHO, 2018) in the childbearing process. Thus, early and consistent ANC attendance especially in the early stages of pregnancy has been identified as crucial in early detection and treatment of maternal health problems in pregnancy and serves as a good basis for proper management during and after childbirth (Manyeh et al., 2020). Yet in Ghana, despite the high antenatal care attendance rate, skilled birth attendance is relatively low (Baatiema et al., 2019). For instance, whilst Ghana achieved 97% coverage of ANC attendance in 2013, skilled health personnel attended only 47.1% of births. Corroborating the findings of Baatiema et al. the 2014 Ghana Demographic and Health Survey (GDHS) showed that while Brong Ahafo achieved 98.9% of ANC attendance, skilled health personnel attended 78.3% of mothers. The remaining 21.6% of mothers still practised home delivery despite the introduction of policies and initiative like safe motherhood (1987) and free-exemption policy for maternal care (2008). This raises questions around the limited evidence on whether antenatal care attendance translates into skilled birth attendance in the Ghanaian health research discourse (Baatiema et al., 2019), and the need to explore and examine other determinants of skilled birth attendance.

This is crucial as several studies conducted in sub-Saharan Africa and different parts of the country have examined the determinants of skilled birth attendance and have provided consistent and mixed results. Baatiema and colleagues referencing studies conducted in Ethiopia and Kenya explained that skilled personnel assisted only 23.5% of respondents. Rural urban disparity was also observed, which turned to favour urban dwellers (Baatiema et al., 2019; Tadese and Ali, 2014). Similar findings were realised in Kenya (Asweto et al., 2014; Baatiema et al., 2019; Gitimu et al., 2015). Overall, the conclusion arrived at is that while majority of women in developing countries utilise antenatal care (ANC) during their pregnancy, their deliveries often lack skilled birth attendants (Manyeh et al., 2017). The authors emphasised that the importance of skilled attendance at delivery is crucial for decreasing maternal and neonatal mortality, yet many women in low and middle-income countries deliver without skilled attendance (Manyeh et al., 2017).

In the specific context of Ghana, studies have examined different dimensions of skilled birth attendance. For instance (Baatiema et al., 2019), examine whether antenatal care attendance translates into skilled birth and concluded that there was higher inclination towards skilled birth attendance among women who had at least four antenatal care visits. In their study, Dickson et al. examined the association between background characteristics and choice of skilled providers of antenatal care services in Ghana, and established that choice of skilled providers of antenatal care services were predicted by some predisposing factors including education, ethnicity, and ecological zone (Dickson et al., 2018). In an examination of the determinants of skilled birth attendants at delivery in rural southern Ghana (Manyeh et al., 2017), reached the conclusion that although 98.29% of the study participants receive antenatal care services during pregnancy, only 68.89% were assisted by skilled persons at their last delivery. Similar conclusions were reached by other studies (Amoakoh-Coleman et al., 2015) including in a multi-country study involving Ghana (Adjiwanou and LeGrand, 2013).

While these previous studies examined ANC and skilled delivery broadly from various dimensions, this study specifically focuses on three key elements of ANC-information, education and communication to assess its influence on prenatal and skilled delivery in Ghana as this has not been the focus of much research in previous studies. This is crucial as evidence from the Tano North District Health Information Management System (DHIMS); the setting of the study showed a different trend about ANC attendance and skilled delivery. The district witnessed a sharp increase in birth deliveries attended by skilled health personnel from 1,979 (79.2%) in 2015 to 2,070 (82.2%) in 2016. In addition, two maternal deaths were recorded in 2016 as against four in 2015. Between the periods, there were further improvements in maternal health figures from 31 in 2015 to 17 in 2016 regarding stillbirths. These positive figures, notwithstanding, the district failed to achieve the 90% target proposed by the United Nations General Assembly as at 2016. However, it was considered an achievement worthy of note.

The study was, therefore, undertaken to assess whether the increase in skilled attendance at deliveries in the Tano North district was as a result of effective IE&C activities following an IE&C programme intervention. IE&C refers to information, education, and communication activities in the form of teaching, messages, posters, flyers, leaflets, brochures, booklets, messages for health education sessions, and all activities that aim at motivating behaviour change for pregnant women towards attending pre-natal, and ANC; and opting for health facility and skilled delivery with the ultimate goal of achieving positive pregnancy outcomes. These include but not limited to information and communication about pregnancy complications, risk, importance of ANC, nutrition during pregnancy, monitoring pregnancy, vaccination, malaria prevention and treatment, HIV/AIDS screening, and health facility and skilled delivery. Overall, information, education and communication initiatives are grounded in the concepts of prevention and primary health care as public health education and communication seek to empower people vis-à-vis their health actions (WHO, 2001). Exposure to IE&C activities above was measured by ANC attendance by the women who participated in this study. This was because various topics and activities of pregnancy mentioned above are usually accessed during ANC. Thus, a pregnant woman was deemed to have enough exposure to IE&C related to pregnancy and childbirth if she attended ANC at least four times prior to delivery.

Thus, this study contributes to our understanding of the relationship between information, education and communication and prenatal and skilled delivery in Ghana using quantitative data collected from pregnant women attending ANCs and those who had delivered in a health facility in the Tano North district of the Brong Ahafo region. Because the focus of the study was to determine the association between IE&C and skilled delivery, a fairly representative sample of women (393) drawn from a total population of 22,733 who attended ANC in selected health facilities from eight weeks of pregnancy onwards were included. This provided rich information on the association between IE&C and skilled birth attendance in the district. The specific objectives of the study were to determine the relationship between IE&C and place of delivery, the proportion of births attended by skilled health personnel and to assess how information, education and communication influenced prenatal and skilled delivery. Again, we sought to examine the topics discussed at ANCs and to determine how mother's knowledge, attitude and perception on prenatal and skilled delivery were influenced by information, education and communication.

## 2 Materials and methods

### 2.1 Design and setting

The study adopted a descriptive cross-sectional quantitative study design that enabled the investigators to collect and examine data at one specific point in time from a sample of women attending ANC and or delivered at selected health facilities in the district. This design was considered appropriate in achieving the objective of the study since the study sought to provide population profiles of the targeted population of interest for a defined time period (Bowling, 2014).

The district is located on the Kumasi-Sunyani trunk road with land size of about 700 square km. The estimated population of the district projected from the 2010 population & Housing Census for 2020 is 101,305 (GSS, 2020) of which children 0–11 months were 3,666 in 2016; Women in Fertility Age [WIFA] 23,454 and expected pregnancy 3,909 in 2017 (GHS, 2018) with a crude birth rate of 28.9% in 2010 (GSS, 2012). It is located to the north west of the Sunyani municipality, Asutifi district to the south west, Ahafo Ano South district (in the Ashanti Region) to the south, Tano south district to the south west and Offinso district (also in Ashanti Region) to the north east.

Its main ethnic groups are Akan, Hausa, Kusasi, Bimobas, Sisalas, Grunshi and Ewe. Majority are Christians, followed by Muslims and few traditional worshippers. The district has one hospital, six health centres, three functional Community based Health Planning Services (CHPS) compounds with Community Health Officers (CHOs) at post and thirty-three (33) demarcated CHPS zones and 126 communities. Eighty-five percent of the roads are untarred which makes it very difficult to render health services like immunisation to the communities.

### 2.2 Sample size determination

Sample size for the study was determined using the formulas by Kothari (2004) and Godden (2004) and the two formulas yielded similar sample size estimation. In addition, a study conducted by Dhakal & Shrestha in Nepal in 2016 found knowledge on birth preparedness and complication readiness as 58.1% among prenatal and skilled delivery women. Hence, a prevalence (P) of % 0.581 was used. Substituting this into the formula by Kothari (2004), a sample size of 374 was recommended. However, to take care of possible drop out of participants, a 5% non-response rate was calculated bringing the sample size to 393 participants. The total population from which the sample size was drawn from the ANC registers of the health facilities included in the study was 22,733.

### 2.3 Sampling method

Purposive and systematic sampling methods were used to select the facilities. The selection of the district hospital, the first referral point in the district, three (3) health centres-second referral point, and two (2) CHPS compounds were based on purposive sampling while a systematic sampling procedure was adopted to select the women from each of the facilities. The target population consisted of all pregnant women who used the ANC and delivered in the district facilities and hence the sample frame was pregnant women selected from the ANC register. A random selection of all pregnant women who attended ANC and delivered at the hospital, health centres and CHPS compounds covering a period of one (1) year in the Tano North district (now Tano North Municipal) was done. Specifically, a simple random sampling was used to randomly select pregnant women using the lottery method. This comprised all pregnant women who visited the district facilities for ANC from 8 weeks up to 36 weeks (9 months).

The inclusion criteria applied consisted of all pregnant women including adolescent pregnant girls who attended antenatal clinic in the district. Those excluded were women who were not pregnant and pregnant women who used the facilities but refused to give consent to participate in the study.

### 2.4 Data collection instruments and procedure

Open-ended and closed-ended questionnaires were used to collect data from pregnant women. The questionnaire was field-tested in a facility that was not part of those sampled. Data were collected on demographic characteristics of participants, relationship between IE&C and place of delivery including the usefulness of each of the different independent variables, topics often discussed at the ANCs among others. Data collection occurred in January 2018. The results have been presented in the form of tables and frequency distribution. Chi-square (for categorical data) was used to test the association between places of delivery and the perception, knowledge and attitude level of mothers on prenatal care and skilled delivery.

### 2.5 Ethical consideration

The study received ethical approval from the Ghana Health Service (GHS) Ethical Review Committee (ERC) with approval number: GHS/RDD/ERC/ADMIN/APP/669. Written informed consent as well as permission to conduct the study were also sought from participants and the Tano North District Health Directorate, health facilities and CHPs compounds selected for the study. To ensure anonymity and confidentiality of participants, identification codes were used in data presentation, analysis and reporting. Also all data were de-identified during data collection and analysis to ensure that personal identifiable information was not collected and/or processed.

## 3 Results

**Figure 1:**
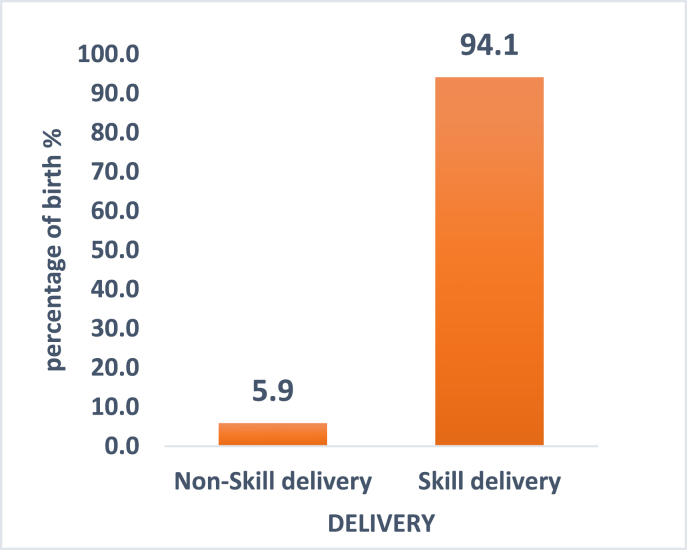
Proportion of birth attended by skilled and non-skilled personnel.

**Figure 2:**
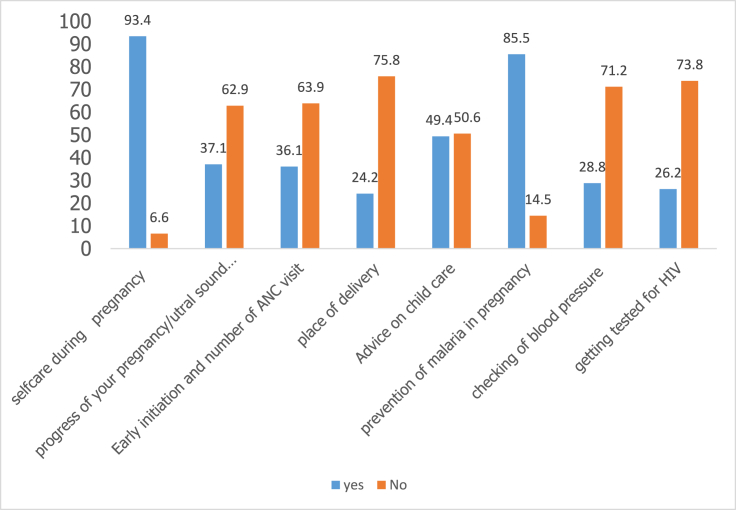
Topics discussed with mothers during Antenatal care.

In the study, 393 mothers were surveyed using questionnaire. The results of the study are presented in six broad sub-headings, which cover the demographic characteristics of participants (mothers), Table 1; the relationship between IE&C and place of delivery, Table 2; the association between demographic characteristic and Odds of being attended by skilled personnel, Table 3; and the association between IE&C and Odds of being attended by skilled personnel, Table 4; the proportion of birth attended by skilled and non-skilled personnel, Figure 1; and the various topics discussed with mothers during antenatal clinic, Figure 2. The demographic characteristics were presented using mean age, standard deviation, age groups, marital status, educational level, occupation, religion, ethnicity, place of delivery and personnel involved, number of birth attended by skilled attendants, number of children delivered including stillbirth, dead or alive and age of baby.

**Table 1:** Demographic Characteristic of mothers.

Variable	Frequency (N)	Percent (%)
**Mean age (S.D)**	27.6 (±6.26)	

Age group		
<20	27	6.9
20–29	209	53.1
30–39	139	35.4
40+	18	4.6
**Marital Status**		
Single	80	20.4
Widow	1	0.2
Divorce/separated	9	2.3
Married	247	62.9
Cohabiting	56	14.3
**Educational Level**		
None	61	15.5
Primary	52	13.2
JHS	172	43.8
SHS	76	19.3
Tertiary	32	8.2
**Occupation**		
Farmers/Fisherman	123	31.3
Self employed	176	44.8
Public servant	32	8.1
Unemployed	62	15.8
**Religion**		
Christian	340	86.5
Muslim	49	12.5
Traditional	4	1.0
**Ethnicity**		
Akan	233	59.3
Ewe	10	2.5
Guan	26	6.6
Ga	4	1.1
Mole-Dagbani	120	30.5

**Table 2:** Pregnancy and delivery characteristics.

Variable	Frequency	Percent
**Antenatal care attendance**		
1–2	159	40.46
3–4	140	35.62
5+	94	23.92
**Place of delivery**		
Hospital	161	40.9
Health center	160	40.7
CHPS	49	12.5
Home	23	5.9
**Delivery type**		
Non-skilled	23	5.9
Skilled	370	94.1
**Home delivery type**		
TBA	18	78.3
Self	2	8.7
Relatives	3	13.0

**Table 3:** Relationship between IE&C and place of delivery.

Variable	Place of delivery					P-value
	Hospital [N = 161]	Health center [N = 160]	CHPs [N = 49]	Home [N = 23]	Chi-square X^2^	
**Information**						
Not useful	94 (58.4)	75 (46.9)	24 (48.9)	2 (8.7)	20.85	0.000
Useful	67 (41.6)	85 (53.1)	25 (51.0)	21 (91.3)		
**Education**						
Not useful	3 (1.9)	2 (1.3)	0 (00.0)	0 (00.0)	1.38	0.711
Useful	158 (98.1)	158 (98.8)	49 (100.0)	23 (100.0)		
**Communication**						
Not useful	3 (1.9)	2 (1.3)	0 (00.0)	0 (00.0)	1.38	0.711
Useful	158 (98.14)	158 (98.75)	49 (100.0)	23 (100.0)		
Overall IE&C						
Not useful	3 (1.86)	2 (1.25)	0 (0.0)	0 (0.0)	1.38	0.711
	158 (98.1)	158 (98.8)	49 (100.0)	23 (100.0)

**Table 4:** Association between demographic characteristic and odds of attendant by skilled personnel.

Variable	Non-Skilled birth attendant	Skilled birth attendant	Chi-square (P-value)	COR (95% CI)P-value
**Age group**				
<20	0 (0.0)	27 (7.3)		
20–29	12 (52.2)	197 (53.3)		0.28 (0.01–4.98) 0.392
30–39	11 (47.8)	128 (34.6)		0.20 (0.01–3.55) 0.275
40+	0 (0.0)	18 (4.9)	3.87 (0.275)	0.67 (0.12–35.43) 0.845
**Marital status**				
Single	2 (8.7)	78 (21.0)	14.73 (0.005)	
Widow	0 (0.0)	1 (0.2)		0.09 (0.00–2.98) 0.181
Divorce/separated	3 (13.0)	6 (1.6)		0.05 (0.00–0.36) 0.002
Married	16 (69.6)	231 (62.9)		0.44 (0.11–1.73) 0.244
Cohabiting	2 (8.7)	54 (14.5)		0.69 (0.11–4.14) 0.689
**Occupation**				
Farmers/Fisherman	17 (73.9)	106 (28.6)	21.78 (0.000)	
Self –employed	3 (13.0)	173 (46.8)		8.14 (2.52–26.32) 0.00
Public servant	0 (0.0)	32 (8.6)		10.68 (0.62–182.52) 0.102
Unemployment	3 (13.1)	59 (16.0)		2.79 (0.84–9.18) 0.091
**Religion**				
Christian	20 (86.9)	320 (86.5)		
Muslim	1 (4.4)	48 (13.0)		2.06 (0.38–11.15) 0.398
Traditionalist	2 (8.7)	2 (0.5)	15.44 (0.000)	0.06 (0.01–0.39) 0.003
**Education**				
None	11 (47.8)	50 (13.5)		
Primary	7 (30.4)	45 (12.2)		1.38 (0.50–3.76) 0.528
JHS	4 (17.4)	168 (45.4)		8.52 (2.74–26.50) 0.000
SHS	1 (4.4)	75 (20.3)		11.46 (2.01–65.19) 0.006
Tertiary	0 (0.0)	32 (8.7)	30.6 (0.000)	14.80 (0.84–259.89) 0.065

From the table, the mean age of participants was 27.6 ± 6.26 with majority of the 393 mothers being in the age group of 20–29 years (209/53.1%). Again, majority of the mothers 247 (62.9%) surveyed in the study were married while Junior High School (JHS) was the educational level attained by majority [172 (43.8%)] of the participants. The findings also revealed that majority 176 (44.8%) of the mothers were self-employed. In terms of religion, Christians were in the majority 340 (86.5%) with Akans as the dominant ethnic group 233 (59.3%).

### 3.1 Delivery characteristics and outcomes

To measure the exposure of the pregnant women to IE&C materials and activities described under materials and methods above, we examined the attendance data of ANC at various health facilities. From Table 2, the number of ANC attendance declined over time. Those who attended 1–2 times were in the majority with 159 (40.46%) and the age group of 21-20 were found in this category, while those who attended 5 + times were 94 (23.92%). This category was characterized by those in the age group of 41–48. An almost equal number of mothers 161 (40.97%) and 160 (40.7%) gave birth at the hospital and the health centre respectively. Three hundred and seventy (94.1%) had skilled delivery while 23 (5.9%) delivered without skilled professional attendance. Of the 23 participants who gave birth at home, majority 18 (78.3%) were delivered by Traditional Birth Attendants (TBA).

### 3.2 The relationship between IE&C and place of delivery

One key objective of this study was to determine the relationship or association between IE&C exposure and place of delivery. Having the right information, being educated and communicated to appropriately is critical to making decisions to attend and continue to attend ANC, and being adequately prepared for the child bearing process including the availability of items needed for delivery are critical for ensuring health facility and skilled delivery. Using the data on ANC attendance and place of delivery, we performed chi square (*X*^*2*^) to determine the relationship between IE & C and place of delivery among the 393 participants in this study. The analysis are based on how useful each component of IE&C was perceived by the participants and whether or not that helped them in their choice of place of delivery. The results are presented in Table 3.

Table 3 shows that of the 161 mothers who delivered at the hospital, 94 (53.4%) viewed the information received during ANC including birth preparedness and complication readiness, items needed by mothers for delivery, financial support from partners and family members as not being useful while 67 (41.6%) felt it was useful. However, of the 160 mothers who delivered at the health centres, majority 85 (53.1%) said the information on birth preparedness and how to care for oneself and the newly born child was useful while 75 (46.9%) said it was not useful. Also, more consensus trends were observed at the CHPS zone, as among the 49 mothers who delivered, 25 (51.0%) said it was useful and 24 (48.9%) said it was not useful. Out of the 23 mothers who delivered at home, only 2 (8.7%) said the information they received was not useful while majority 21 (91.3%) said the information was useful. There was a significant association between information and place of delivery (X^2^ = 20.85, P = 0.000 α = 0.05). However, there was no significant association between the information and how it was communicated in relation to place of delivery (X^2^ = 1.38, P = 0.711 α = 0.05). Out of the 161 mothers who delivered at the hospital, majority 158 (98.1%) of the mothers said how the information on birth preparedness and how to care for oneself and the newly born child was communicated through focus group and one-on-one discussion during ANC were useful while 3 (1.9%) thought otherwise. A more positive view of how the information was communicated was seen across delivering at health centres, CHPS compounds and even at home. This is interesting as these are the levels where primary health care (PHC) occurs. Of 160 mothers who delivered at the health centres, 158 (98.8%) said the communication was useful with only 2 (1.3%) disagreeing it was important; all 49 (100.0%) mothers who delivered in the CHPS compounds said the communication was useful. Hundred percent of the mothers (23) who delivered at home were of similar views that the communication received during the ANCs was useful in preparing them adequately for delivery.

From the presentation, it is apparent that there was no significant association between education and place of delivery (X^2^ = 1.38, P = 0.711 α = 0.05). Majority 158 (98.1%) of the mothers who delivered at the hospital said the education they received during ANC sessions including self-care during pregnancy, breastfeeding and early initiation to ANC and prevention of malaria were useful while 3 (1.9%) said it was not useful. Moreover, 158 (98.8%) of mothers who delivered at the health centre said the education they received during ANC was useful while 2 (1.3%) said it was not useful. Among mothers who delivered in the CHPS Compounds, majority 49 (100%) said the education they received during ANC was useful while none of them said it was not useful. Again, 23 (100%) of the mothers who delivered at home said the education received was useful even though they did not deliver at the facilities.

### 3.3 Association between demographic characteristic and odds of being attended by skilled personnel

Another key objective of the study was to determine whether an association existed between the demographic characteristics of mothers and the odds of being attended by a skilled personnel. The results are presented in Table 4. Analysis of the survey data revealed that majority of the women who had skilled delivery were younger compared to those women who did not have skilled delivery. In Table 3, 197 (53.3%) of the women in the age group 20–29 received skilled delivery compared to 12 women in that same category who did not have skilled delivery. On the other hand, 128 (34.6%) of those who received skilled delivery were between ages 30–39 years. Chi square analysis, however, showed that there was no statistically significant association between age group and type of delivery among the participants in this study (χ2 = 3.87, p = 0.275).

In terms of marital status, we found a statistically significant association between marital status and accessing skilled delivery. In the analysis, 231(62.9%), 78(21%), and 54 (14.5%) who received skilled attendance were married, single, and those who cohabited with their spouses respectively. Although the analysis show more married women received skilled delivery compared to other women, the *X*^*2*^ analysis showed that single women were more likely than other women to have their delivery attended by skilled professionals. Like marital status, we found that a statistically significant association existed between mothers' occupation and odds of being attended by skilled birth attendant (χ2 = 21.78, p = 0.000). From Table 3, majority [173 (46.8%)] of those who delivered with skilled birth attendants were self-employed. This was followed by 59 (16%) public servants and 32 (8.6%) unemployed women. We also found that women employed were more likely than unemployed women to access skilled delivery as those in formal or self-employment were 10.7 times likely to be attended to by skilled birth attendants than unemployed women.

With regards to religious affiliation and accessing skilled delivery, we found that the large majority of the women were Christians and so formed majority for both skilled [320 (86.5%)] and unskilled (20) delivery. However, X^2^ analysis revealed a statistically significant association between religion and skilled delivery among the women in this study with (χ2 = 15.44, p = 0.000) and COR = 0.06, 95% CI (0.01–0.39) and this was statistically significant p = 0.003.

Above all, a significant association between the mother's level of education and being delivered by a skilled birth attendant (χ2 = 30.6, p = 0.000) was observed in the study. Out of the 23 mothers who were delivered by non-skilled birth attendants, most 11 (47.8%) of them had no formal education. Conversely, a greater proportion 168 (45.4%) of the mothers delivered by skilled birth attendants attained at least JHS education. Evidently, mothers who attained SHS were 11.46 times more likely to be delivered by skilled birth attendant compared to those without formal education COR = 11.46, 95% CI (2.01–65.19) p = 0.006 indicating a statistically significant relationship. Educated mothers have more opportunities to work, get well-paid jobs to cater for their health issues compared to mothers who are uneducated, and may have to depend solely on their husbands when seeking health care.

### 3.4 Proportion of birth attended by skilled and non-skilled personnel

The results of the proportion of births attended by skilled and non-skilled personnel are presented in the figure below. Out of the 393 mothers surveyed, majority 370 (94.1%) of them were delivered by skilled health personnel while 23 (5.9%) were delivered by non-skilled personnel.

### 3.5 Association between IE&C and odds of attendance by skilled personnel

In our attempt to determine whether the information, education and communication mothers received at ANC had any influence on being attended by a skilled birth personnel, mothers were asked how useful they found that in relation to birth preparedness and complication readiness, acquisition of pre-delivery items, financial support and availability of transportation for referral among others. The results are presented in Table 5.

**Table 5:** Association between IE&C and Odds of attendance by skilled personnel.

Variable	Non-Skilled birth attendant	Skilled birth attendant	Chi-square (P-value)	COR (95% CI)P-value
**Information**				
Not useful	2 (8.7)	193 (52.2)		
Useful	21 (91.3)	177 (47.8)	16.36 (0.000)	0.10 (0.02–0.40) 0.001
**Education**				
Not useful	0 (0.0)	5 (1.4)		
Useful	23 (100.0)	365 (98.6)	0.31 (0.575)	1.41 (0.07–26.34) 0.816
**Communication**				
Not useful	0 (0.0)	5 (1.4)		
Useful	23 (100.0)	365 (98.6)	0.31 (0.575)	1.41 (0.07–26.34) 0.816

Of the 23 mothers delivered by non-skilled birth attendants, majority 21 (91.3%) said the information received at ANCs on topics such as birth preparedness and complication readiness, items needed by mothers for delivery, financial support from partners and family members and availability of transportation for referral from one sub-district to another was useful while 2 (8.7%) felt otherwise. Surprisingly, of 370 mothers delivered by skilled birth attendants, majority 193 (52.2%) thought such information was not useful with nearly about a greater number 177 (47.8) saying it was useful. Chi-Square analysis revealed a significant association between information mothers received and odds of being attended by skilled birth attendants; again demonstrating a statistically significant association (χ2 = 16.36, p = 0.000). COR = 0.10, 95% CI (0.02–0.40) p = 0.001.

With regards to the education and communication mothers received at ANCs and odds of being attended by skilled birth attendants, no significant association was observed. Hundred percent (23) of the women delivered by non-skilled birth attendants said the education they received including self-care during pregnancy, prevention of malaria, progress of their pregnancy/ultra sound scan and how they were communicated during ANC were useful. Similarly, majority 365 (98.6%) of the 370 women delivered by skilled birth attendants said the education they received and how it was communicated were useful. Thus Chi-Square computation (χ2 = 0.310, p = 0.575). COR = 1.41, 95% CI (0.07–26.34) and this was not statistically significant p = 0.816.

### 3.6 Topics discussed with mothers during antenatal care

Another task in the study was to find out from the women some of the main topics of focus during ANCs. Getting a sense of some of the issues discussed were considered crucial to understanding the significance of the information, education and how it was communicated to the women and the extent to which it influenced place of delivery and by skilled birth attendants.

Analysis of the questionnaire revealed that topics often discussed included: self-care during pregnancy, malaria prevention during pregnancy, place of delivery, the importance of getting tested for HIV/AIDS, early initiation of breastfeeding, progress of pregnancy/ultra sound among others. The results are presented in the below figure.

Majority 367 (93.4%) of the 393 women were educated on self-care during pregnancy; malaria prevention during pregnancy 336 (85.5%); place of delivery 95 (24.2%); the importance of getting tested for HIV/AIDS 103 (26.2%) among others. However, majority 298 (75.8%) of the mothers were not educated on place of delivery as it was mostly restricted to mothers with suspected complicated conditions so they could see specialists or medical experts. Similarly, majority 290 (73.8%) of the mothers were not educated on HIV even though they all testified that they were tested before delivery. The story was the same with early initiation of breastfeeding and number of ANC visits as only 142 (36.1%) were educated with 251 (63.9%) of the mothers saying they were not. This trend was also seen in progress of pregnancy/ultra sound scan with majority 247 (62.9%) not being educated. While mothers with older babies could not tell whether they were educated on the progress of their pregnancy/ultra-sound scan, there were able to confirm that they had ultra sound scan before delivery. On malaria prevention, mothers of older babies involved were not able to recall whether they were educated or not but they were able to testify that there were given SP (sulphurdoxine pyrimethamine).

## 4 Discussion

The general aim of this study was to determine the influence of information, education and communication on prenatal and skilled delivery in the Tano North district of the Brong Ahafo region. The results of this study show that there are important links between effective information, education and communication and skilled delivery. As recommended by the World Health Organisation's new antenatal care model, IE&C require time. This implies that the more effective IE&C conducted by health personnel during ANC, the better the encouragement to women to deliver at the various health facilities thereby increasing attendance by skilled personnel.

It is revealed that 94.1% of the mothers who participated in the study were delivered or attended by skilled health personnel in their recent deliveries. This finding is higher than what was reported in the latest (2014) Ghana Demographic and Health Survey, and above the regional figure where 78.3% of mothers were attended by skilled health personnel in the then Brong Ahafo Region (GSS, 2015). Again, it is higher than what was reported in the Tano North District Health Information Management System for 2016 (82.2%), but lower than the results in the Ghana special demographic and health survey conducted in 2017, where ANC coverage by a skilled provider slightly improved to 98% (GSS et al., 2018), which demonstrates improvement over the years. Elsewhere in Sub-Saharan Africa and Asia, the finding is not consistent with a number of studies. In a study conducted in Ethiopia to determine the prevalence of skilled birth attendant utilisation and its correlates, a rather low (18.77%) birth attendance by skilled birth attendants was reported (Alemayehu and Mekonnen, 2015). Gitimu and colleagues also reported in their study that only 49.3% of the mothers were attended by skilled birth attendants (Gitimu et al., 2015) in Kenya. Similarly, a study on skilled birth attendance in Bangladesh showed a lower (35.9%) proportion of births by skilled birth personnel (Al Kibria et al., 2017). The high proportion of births attended by skilled birth attendants in this study might be due to the fact that most of the mothers were literate and there was a significant association between mothers' level of education and being attended by skilled birth attendants (χ2 = 30.6, p = 0.000). This is in keeping with a study conducted in Ghana where the authors observed that skilled birth attendance was predicted by some predisposing factors including education, ethnicity, and ecological zone (Dickson et al., 2018).

Also, majority of the mothers' had good attitude towards antenatal and health facility delivery than home delivery that made them neglect any bad cultural practices that would have hindered their decision not to deliver at the health facilities. Again, the district had more experienced midwives that had served for almost four (4) to eleven (11) years. So they were able to provide detailed information to mothers during antenatal sessions including birth preparedness and complication readiness, items needed by mothers for delivery, financial support from partners and family members and availability of transportation for referral from one sub-district to another.

In this study, there was a significant association between the mother's level of education and being attended by a skilled personnel (χ2 = 30.6, p = 0.000) and when mother's level of education increases the higher the odds of being delivered by skilled birth attendants. Mothers who attained SHS were 11.46 times more likely to be delivered by skilled birth attendant compared to those without formal education COR = 11.46, 95% (2.01–65.19) and this was statistically significant p = 0.006. This finding is consistent with findings by Al Kibria and his colleagues in Bangladesh, who reported that educational level was positively associated with deliveries attended by skilled birth attendants (Al Kibria et al., 2017). It is also consistent with findings by Alemayehu and Mekonnen (2015) in Ethiopia who found that educational status of mothers was also significantly associated with the utilisation of skilled birth attendants. Mothers who had attained primary and secondary education and above were more likely to utilise skilled birth attendants than those mothers who were unable to read and write (Alemayehu and Mekonnen, 2015). The association between educational status of a mother and skilled birth attendance might be due to greater knowledge and awareness of the importance of skilled birth attendants in deliveries. The association of education level may also be linked to the wealth quintile, as educated people have more opportunities to work and get well-paying jobs. Moreover, education was the strongest and most consistent predictor of health status.

We also found a significant association between information received by pregnant women and place of delivery (X^2^ = 20.85, P = 0.000 α = 0.05) in that the usefulness of information to mothers influenced their choice of health facility delivery. For instance, among the 160 mothers who delivered at the health centres, majority 85 (53.1%) and 51% of those who delivered at CHPS compounds said they received useful information during ANC that influenced their choice of place of delivery. The association between information and place of delivery in this study might be due to the fact that the district had more experienced midwives in all the facilities who are able to provide detailed information about male involvement, birth preparedness and complication readiness towards ANC and delivery through community participation. It is, therefore, clear that information provided by nurses and other healthcare professionals during ANC visits is critical in educating and motivating pregnant women to seek health facility and skilled delivery. These findings, therefore, underscores the importance of ANC as an engine of IE&C as it has been argued that skilled attendance at birth is an effective intervention to reduce maternal and early neonatal morbidity and mortality (Shiferaw and Modiba, 2020).

A yet critical finding in this study was the relationship between the demographic variable of being married and place of delivery and being attended by skilled birth personnel. Marital status was strongly correlated to being attended by skilled birth attendant (χ2 = 14.73, p = 0.005) as 0.4 times of mothers who were married were more likely to be attended by skilled birth attendants as compared to those that are single. COR = 0.005, 95% CI (0.00–0.36) and this was statistically significant p = 0.002. The conclusion reached is that most married women have financial support from their husbands to be attended by skilled birth attendants compared to single women. This is consistent with finding by researchers in Ethiopia that involvement of spouses or sexual partners among other factors was significantly associated with access to skilled delivery (Wilunda et al., 2015). It is also similar with a finding in Sierra Leone that marital status, and especially being married, among other factors was strongly associated with uptake of child delivery services among pregnant women (Tsawe and Susuman, 2020). The finding is, however, inconsistent with other studies that found marital status to have no effect on skilled and place of delivery but found that socio-demographic characteristics discussed above to significantly predict skilled and place of delivery (Esena and Sappor, 2013; Gudu and Addo, 2017; Manyeh et al., 2017).

Another demographic variable in the study that was significantly associated with place of delivery and skilled birth attendance was religion. The X^2^ analysis revealed a statistically significant association between religion and skilled delivery among the women in this study with (χ2 = 15.44, p = 0.000) and COR = 0.06, 95% CI (0.01–0.39) and this was statistically significant p = 0.003. This implies that health authorities in Ghana need to intensify maternal and child health education through religious institutions if increases in skilled birth attendance is to be achieved.

## 5 Limitations

While important findings have been made in the study as seen above, it is acknowledged that the study was subjected to recall bias because most mothers found it difficult to remember information that was received during their ANC sessions. Also, given that this study was conducted in few public health facilities in one district in a region, it makes it difficult to generalise about the whole country due to different contextual variables. This is more so as public and private health delivery set up and access varies.

## 6 Conclusion

The study revealed that there were high proportion of deliveries conducted by skilled birth attendants in the Tano North District because mothers had good attitude towards ANC and delivery as compared to home delivery. Again, this study established that the educational level of the mother and marital status had significant association with skilled birth attendants, as mothers who attained higher educational levels were more likely to be attended by skilled birth attendants compared to uneducated mothers; as well as being financially supported by partners and family members. Moreover, this study found that information mothers received during ANC had influence on place of delivery with a significant association. However, overall information, education and communication (IE&C) had a rather low influence on ANC and delivery. Other factors such as age, education, marital status, religion and socio-economic status were stronger predictors of skilled delivery. In order to improve ANCs and skilled birth attendance, this study suggests the incorporation of IE&C into nursing training curriculum to orient students on the importance of the programme. In addition, we recommend that policies should be developed in relation to incorporating traditional birth attendance in the primary health care system since majority of those who delivered at home were attended by the TBAs. Also, effective supervision of IE&C during ANC sessions to ensure the WHO new recommended antenatal care model is achieved in prenatal and skilled delivery is recommended.

## Declarations

### Author contribution statement

Evelyn Amponsah: Conceived and designed the experiments; Performed the experiments; Analyzed and interpreted the data; Contributed reagents, materials, analysis tools or data.

Adam Fusheini: Conceived and designed the experiments; Performed the experiments; Analyzed and interpreted the data; Contributed reagents, materials, analysis tools or data; Wrote the paper.

Awolu Adam: Performed the experiments; Analyzed and interpreted the data.

### Funding statement

This research did not receive any specific grant from funding agencies in the public, commercial, or not-for-profit sectors.

### Data availability statement

Data included in article.

### Declaration of interests statement

The authors declare no conflict of interest.

### Additional information

No additional information is available for this paper.
